# The General Nursing Council for England and Wales

**Published:** 1921-04-02

**Authors:** 


### THE GENERAL NURSING COUNCIL FOR ENGLAND AND WALES.

The thirteenth meeting of the General Nursing Council was held on Thursday, March 24, at the Ministry of Health. The agenda paper records, in a concluding note, that "The Chairman apologises for calling the meeting at such short notice, but so much time has been wasted since February 2 that it was felt to be highly desirable to hold a meeting before Easter to consider the position created by the Law Officers' opinion rather than postpone it until after the Easter vacation." The Chairman of the Council (Mr. J. C. Priestley, K.C.) presided, and those present were: Rev. G. B. Cronshaw, Dr. E. W. Goodall, Dr. Bedford Pierce, Sir T. Jenner Verrall, Miss Cattell, Mr. Christian, Miss Coulton, Miss Cox Davies, Miss Dowbiggin, Mrs. Bedford Fenwick, Miss Lloyd Still, Miss MacCallum, Miss Macdonald, Miss Sparshott, Miss Suiss, and Miss Worsley. Letters of regret had been received from Miss Tuke, the Hon. Mrs. Eustace Hills, and Miss Seymour Yapp, the latter being prevented by illness from attending.

### Minutes of Last Meeting.

The Chairman brought up a question as to the correctness of one of the minutes of the last meeting on which he desired the Council's view. Sir Jenner Verrall, who raised the question originally, explained his view, and suggested that it should be a rule that where an amendment is not seconded it shall not be recorded on the minutes. After some discussion the minute was adopted, and Sir Jenner Verrall's suggestion, put to the Council in the form of a resolution, was negatived. Miss Cattell desired that the minutes should be amended to include the vote of thanks to the Chairman, which was proposed and seconded at the last meeting but had not been recorded. The Chairman expressed his acknowledgments of this desire, and promised that the vote of thanks should be duly recorded.

Letters were submitted (1) from Mrs. Paterson, St. Bernard's Crescent, Edinburgh, expressing intense regret that the General Nursing Council, to which nurses are looking for assistance, has decided to withdraw nurses from the Labour Ministry's Hours of Employment Bill, which, she states, is just such a Bill as is wanted; (2) from Miss Hett, Joint Honorary Secretary of the Central Council for District Nursing in London, sending that Council's resolution of February 28, which points out the large amount of additional instruction needed to equip a trained nurse for district work, such as special training in tuberculosis work and in venereal diseases, and desires that the attention of the training-schools shall be called to this need; the letter was referred to the Education and Examination Committee; (3) from Miss MacCallum, Honorary Secretary of the Professional Union of Trained Nurses, forwarding the unanimous resolution of a meeting of free members desiring to be included in the Hours of Employment Bill No. 2 to be introduced into the House of Commons by the Minister of Labour; (4) from the Secretary of the Nursing and Midwifery Conference and Exhibition, calling attention to the forthcoming conference and asking for suggestions for subjects and speakers, together with the presence of two or three speakers representing the Council to expound the Registration Act and matters arising out of it. It was decided, on Mrs. Fenwick's motion, that the request should be refused, as it is not considered desirable that the Council, as a statutory body, shall associate itself with an outside organisation; (5) from Miss Margaret Breay, Honorary Secretary of the Registered Nurses' Parliamentary Council, sending a *précis* of the discussion at a meeting held on February 26, when the desirability of providing in the rules for equivalent standards of registration between the three Nursing Councils was urged.

### Discussions in Camera.

The next item on the agenda was to consider the situation created by the opinion of the Law Officers of the Crown, and whether the same should be discussed *in camera*. This opinion had been previously circulated amongst the Council. The Chairman proposed, and Dr. Goodall seconded, that the discussion should be *in camera*. Miss MacCallum, though not opposing the resolution, registered her objection to the principle, and was supported by Mrs. Bedford Fenwick, who holds that the less the Council's proceedings are *in camera* the better. Her wish is that if the Council comes to the conclusion that the rules as approved are not to the best benefit of the profession, then the nurses shall have the whole case placed before them. The resolution was carried, and on Miss Cox Davies' suggestion the discussion on this matter was deferred to the end of the proceedings so as to study as far as possible the convenience of the members of the Press present.

### Recognition of Training in Women's Hospital.

Mrs. Bedford Fenwick then presented the report of the Registration Committee, a lengthy document which was not available to the Press, concerning alterations or amendments of draft rules. These and the discussions thereon it was quite impossible to follow without the documents. The Chairman reported a meeting he had had with gentlemen from Scotland, and Sir E. Coey Bigger from Ireland, concerning the question of the country of original registration. This the other Councils have decided shall be the country of training, whilst the English Council takes the view that the nurse shall be free to register in the country where she desires to practise. The Chairman believes that both Councils will give way to the English Council on the question. The report of the Registration Committee was adopted. It includes provision in the rules for the admission to the general part of the Register of an applicant with not less than two years' training in a hospital for women, followed by one year's training in a Poor-law infirmary or general hospital approved by the Council prior to November 1919. This follows the lines approved by the Council for the admission to the general register of sick children's and fever nurses.

### Sale of Draft Syllabus.

Miss Lloyd Still presented the report of the Education and Examination Committee, which also was not supplied to the Press. The recommendations of the Committee included the form of evidence as to training; subjects of examination and the period and time of examinations; arrangements for the conferences on April 28, with the subject-matter to be brought forward; the sale for 6d. of the Draft Syllabus for training which has been drawn up by the Council. A letter was read from Miss Seymour Yapp, questioning certain details in the recommendations, which was adjourned for consideration by the Education and Examination Committee. A point raised by Miss Villiers (who was not present) was also discussed. The report was adopted.

### New Headquarters Nearing Completion.

Sir T. Jenner Verrall presented the report of the Finance Committee, which was adopted. Sanction was given for expenditure on furniture and office fittings for the new headquarters of the Council in York Gate, for the acceptance of an estimate for painting, and for the purchase of any necessary things to finish the furnishing and equipment of the premises, which the Council formally took over on March 1, 1921. It was agreed to accept a revised estimate for the registration certificate involving an extra £2 per thousand copies, making the cost £95 instead of £75 per 10,000.

Dr. Bedford Pierce presented the report of the Mental Nursing Committee. It was agreed to accept the syllabus of the Medico-Psychological Association as the Council's syllabus of training for the next three years—*i.e.*, till July 1924—in order to bridge over the gap from now till the first State examination in July 1924.

The Council then proceeded to discuss *in camera* the opinion of the Law Officers of the Crown, which had been deferred from No. 4 on the agenda.

